# Bioremediation of soil contaminated crude oil by *Agaricomycetes*

**DOI:** 10.1186/s40201-016-0263-x

**Published:** 2017-03-18

**Authors:** M. Maryam Mohammadi-Sichani, M. Mazaheri Assadi, A. Farazmand, M. Kianirad, A. M. Ahadi, H. Hadian Ghahderijani

**Affiliations:** 1 Department of Biotechnology, Iranian Research Organization for Science & Technology (IROST), Tehran, Iran; 2 Department of Genetic, Faculty of Science, Shahrekord University, Shahrekord, Iran; 3 Isfahan Refinery Environmental Expert, Isfahan, Iran

**Keywords:** Bioremediation, Spent mushroom compost, Crude oil, Pollution, Petroleum contaminated soil

## Abstract

**Background:**

One of the most important environmental problems is the decontamination of petroleum hydrocarbons polluted soil, particularly in the oil-rich country. Bioremediation is the most effective way to remove these pollutants in the soil.

Spent mushroom compost has great ability to decompose lignin-like pollution. The purpose of this study was the bioremediation of soil contaminated with crude oil by an *Agaricomycetes*.

**Methods:**

Soil sample amended with spent mushroom compost into 3%, 5% and 10% (w/w) with or without fertilizer. Ecotoxicity germination test was conducted with *Lipidium sativa*.

**Results:**

The amplified fragment (18 s rDNA) sequence of *this* mushroom confirmed that the strain belonged to *Pleurotus ostreatus* species with complete homology (100% identity). All tests experiment sets were effective at supporting the degradation of petroleum hydrocarbons contaminated soil after three months. Petroleum contaminated soil amended with Spent mushroom compost 10% and fertilizer removed 64.7% of total petroleum hydrocarbons compared control. The germination index (%) in ecotoxicity tests ranged from 60.4 to 93.8%.

**Conclusions:**

This showed that the petroleum hydrocarbons contaminated soil amended with 10% Spent mushroom compost had higher bioremediation ability and reduced soil toxicity in less than three months.

## Background

Contamination of soil by petroleum hydrocarbons is a serious problem in oil producing counties. Release of petroleum into our environment is a main cause of soil and ground water pollution. Soil contamination with petroleum hydrocarbons affects on plants, animals and humans life [1]. Bioremediation is a very effective approach to remove numerous pollutants from many contaminated site. Mycoremediation is defined as a natural or artificial process in which fungi processes are used to degrade contaminants to less toxic or nontoxic forms, thereby reducing or eliminating environmental contamination [2–4]. Ligninolytic fungi (white rot fungi) are able to degradation of petroleum hydrocarbons by extracellular lignin modifying enzymes. These enzymes have very low substrate specify, making them suitable for degradation of a wide range of highly recalcitrant compounds that is structurally similar to lignin. The ligninolytic enzymes consist of lignin peroxidase, manganese peroxidase and laccase [5, 6]. The Spent Mushroom Compost (SMC) contains a consortium of hydrocarbon degrading bacteria and ligninolytic fungi. The SMC contains large amounts of different types of ligninolytic enzymes. It seems that SMC can be effective in the degradiation of petroleum hydrocarbone [7]. Spent mushroom compost is the residual compost waste generated by the mushroom production industry. The possibility of degradation of contaminants, not requiring specialized equipment and the possibility of in situ and ex situ are considered as the advantages of mycoremediation by SMC. The *Pleurotus* genus belonging to the order Agaricales is regarded as one of the commercially important edible mushrooms throughout the world. Species of *Pleurotus* are generally called Oyster mushrooms have been applied to break down the various organic contaminants and have important biotechnology and environmental applications [8, 9].

The aim of this study which is different from previous researches includes the biodegradation potential of SMC of an *Agaricomycetes* to decontamination of petroleum hydrocarbons polluted soils by inexpensive and environmental friendly method.

## Methods

A strain of unidentified *Agaricomycetes* was previously isolated from solid waste of mix culture a mushroom growing factory and designated as MFN (unpublished data) was used in this experiment. Mycelia of isolated fungi cultured on potato dextrose agar (PDA) medium. The spawns of MFN were fruited on the pasteurized sawdust substrate. This substrate, after harvesting the mushroom fruits became the SMC and used for bioremediation process of this study.

Petroleum contaminated soil was collected from Isfahan Refinery were refrigerated in 4 °C until tested [10]. The physicochemical properties of the soil such as pH, moisture, salinity, electrical conductivity and CHNS elemental contents were determined [11].

### Microcosm set-up

About 100gr of contaminated soil was shed in plastic pots. The SMC were added into soil samples in 3%, 5% and 10% (w/w). Since petroleum hydrocarbons have not enough nitrogen, sulfur and phosphorus for microbial growth, therefore urea, ammonium nitrate and K_2_HPO_4_ (0.7, 1, 0.3 g per 10 g soil) were added to some of soil samples [12, 13]. Seven experimental sets were prepared (Table 1). One of the pots was not amended with SMC to serve as control.

**Table 1:** Experimental design

Experimental set	Test experiment
Set A	Petroleum contaminated soil + SMC 3%
Set B	Petroleum contaminated soil + SMC 3% + NPK
Set C	Petroleum contaminated soil + SMC 5%
Set D	Petroleum contaminated soil + SMC 5% + NPK
Set E	Petroleum contaminated soil + SMC 10%
Set F	Petroleum contaminated soil + SMC 10% + NPK
Control	Petroleum contaminated soil + pasteurized sawdust 10%

The pots were labeled and placed at 25 °C. After a period of one, two and three months, soil samples (1 gr) from each vessel were extracted with dichloromethane. The pooled organic phases were dried in room temperature. The residues were dissolved in 5 ml dichloromethane and optical density of each sample was measured by spectrophotometer at 495 nm [14].

### Ecotoxicity test

*L.sativa* is a fast growing, edible herb that is fairy sensitive to toxic chemicals. Germination test were conducted by placing fifty seeds of *Lipidium sativa* in Petri dishes covered with a Whatman No.1 filter paper moisture with soil extracts samples. Control filter-paper plate wetted with distilled water. The plates were incubated at 22 °C for 7 days. The number of germinated seed was counted [15, 16]. All the experiments were carried out in three replicates. The germination index (%) was calculated as follow:

$$ \begin{array}{l}\%\ \mathrm{Germination} = \mathrm{number}\ \mathrm{of}\ \mathrm{germinated}\ \mathrm{seeds}\ \mathrm{in}\ \mathrm{contaminated}\ \mathrm{test}\ \mathrm{soil}/\ \mathrm{number}\ \mathrm{of}\ \mathrm{germinated}\ \mathrm{seeds}\ \mathrm{in}\ \mathrm{control}\\ {} \times 100\end{array} $$

### DNA extraction

Total genomic DNA was extracted as described by Graham et al. About 100 mg fungal mycelia was ground to a fine powder in liquid nitrogen for 10 min and transferred into a 1.5 ml microfuge tube. Lysis buffer (0.25 mM Tris–HCl, 100 mM NaCl, 50 mM EDTA, 0.5% sodium dodecyl sulfate (SDS) was then added. After incubation at 25 °C for 15 min, CTAB buffer (2% CTAB [w/v], 100 mM Tris–HCl, 50 mM EDTA, 1.4 M NaCl) was added. The mixture was placed in room temperature for 15 min. The solution was extracted with phenol: chloroform: isoamyl alcohol (25: 24: 1, v/v), centrifuged at 12000 rpm for 15 min in 4 °C. The upper phase was transferred to new tube, and DNA was precipitated with 0.75 volume isopropanol, washed with 70% ethanol three times, dried, and resuspended in 100 μl TE buffer (10 mM Tris–HCl, 1 mM EDTA, pH 8.0). Genomic DNA was visualized in 1% agarose gel.

### PCR amplification

Amplification and sequencing of used fungi was performed using pairs of 18 s rDNA, FPLFL (5′ ~ GAAAGAGAGTTAAACAGTACG ~ 3′) RPLFL (5′ ~ AGTCTTTCGCCCCTATACC ~ 3′) that their product was 540-566 bp. These primers were designed for PCR detection of reliable species of *Pleurotus spp*. The amplification was carried out in a thermocycler (Eppendorf, Germany) in a 25 μl reaction mixture containing 2.5 μl 10 x PCR buffer, 0.75 μl 50 mM MgCl_2_, 0.5 μL dNTP Mix (10 mM each), 1 μL 10 μM each primer, 1 μl template DNA, 0.2 μl Taq DNA polymerase (5U/μl) and 18 μl nuclease free water. The reaction mixtures were denatured at 96 °C for 6 min and subjected to 35 cycles of 45 s at 94 °C, 40 s at 50 °C, 50 s at 72 °C, and a final extension step of 5 min at 72 °C. Electrophoresis of PCR product was performed at 70v, 390 mA in 1.0% agarose gel and stained with DNA Green Viewer. The PCR products were sent to FAZA Biotech Company for DNA sequencing. DNA sequences were visualized by Chromas version 2.1.1and was submitted to NCBI database (www.ncbi.nlm.nih.gov/Blast) to perform sequence alignment using nucleotide BLAST (BLASTN) [17, 18].

## Results

**Fig. 1:**
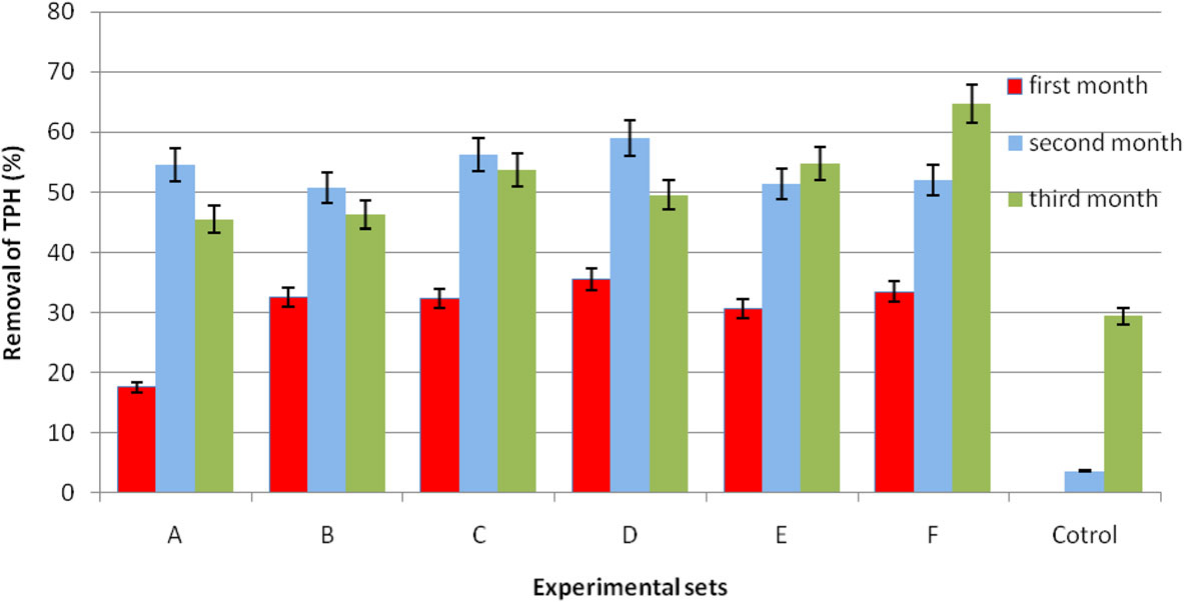
Percentage biodegradation of petroleum hydrocarbon in soil contaminated with SMC of *Pleurotus spp.*

The physicochemical properties of the soil are shown in Table 2. The results of the petroleum hydrocarbons reduction in contaminated soil that were treated with varying amounts of SMC were shown in Fig. 1.

**Table 2:** The physicochemical properties of the soil

Parameters	Soil
pH	7.6 ± 0.1
Moisture	1.3%
Carbon (%)	44.5 ± 0.0
Nitrogen (%)	0.00
Hydrogen (%)	1.78 ± 0.00
Sulfur (%)	0.32 ± 0.03
Sal	3.0 ± 0.1
Electrical conductivity(mS)	5.7 ± 0.1

**Fig. 2:**
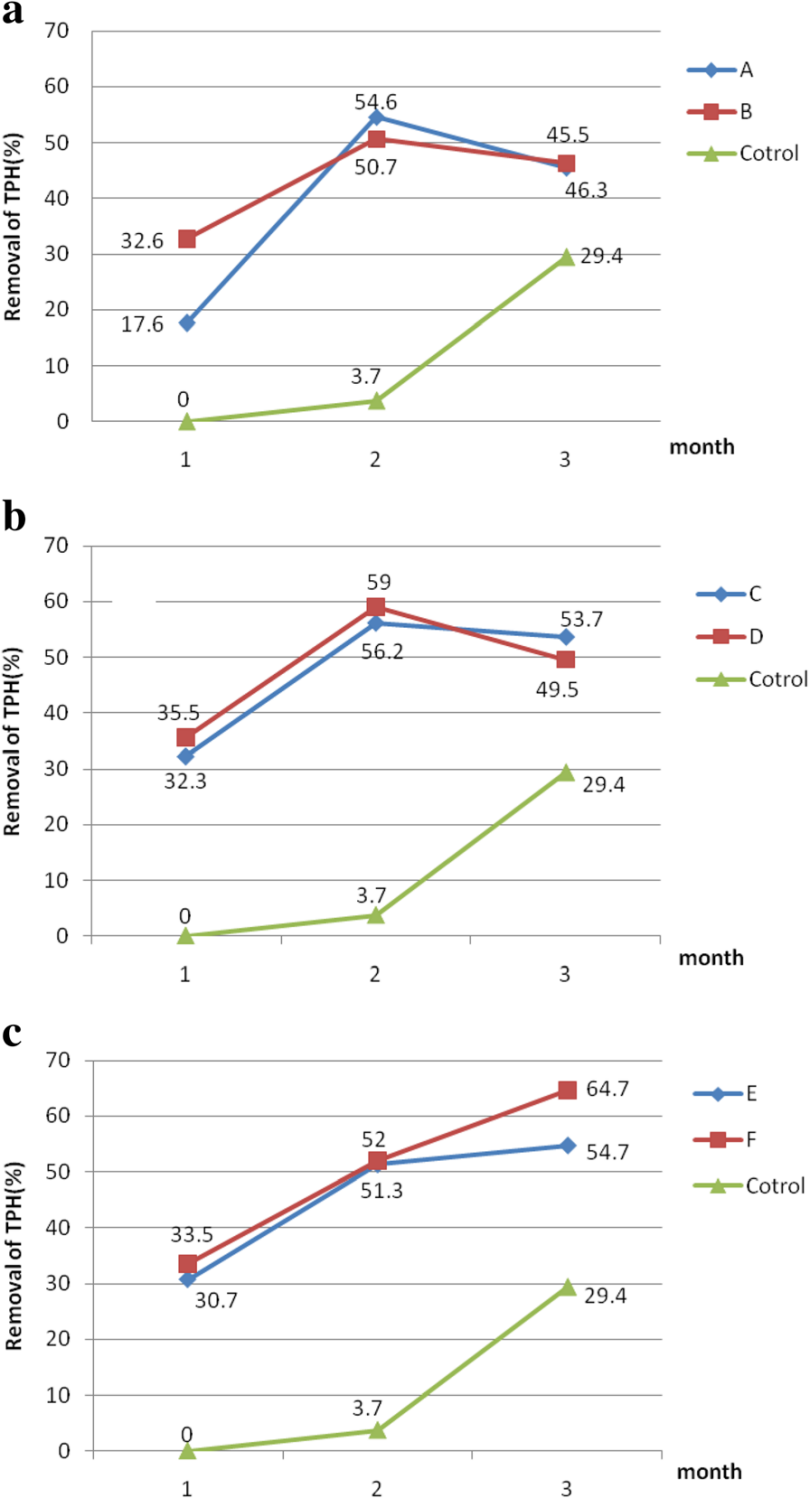
Net Percentage removal of Total Petroleum Hydrocarbon in Soil contaminated with used SMC (**a**: Experimental set A, B; **b**: Experimental set C, D; **c**: Experimental set E, F)

All tests experiment mixtures were effective at supporting the degradation of petroleum hydrocarbons contaminated soil, while the control was not. The percentage biodegradation of petroleum hydrocarbon was calculated by dividing the difference in optical density of samples between the zero time and final by optical density of zero time × 100 in the each case. Statistical analysis indicated a significant difference at *P* < 0.001 between the experimental set A-F and control pot, thus proving the efficient role of SMC to the mycoremediation of petroleum hydrocarbons contaminated soil. Percentage removal of petroleum hydrocarbons contaminate soil in each experimental series were shown in Fig. 2(a-c).

**Fig. 3:**
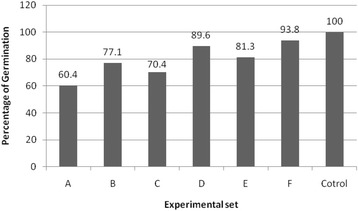
Percentage of germination for all experimental sets (A-F)

The results of the seed germination show in Fig. 3. This clearly shows the effect of active biodegradation in decrease of toxicity of contaminated soil for *L.sativa*. The toxicity of non-treated soil was much higher than that of different treated soil.

The germination index (%) in ecotoxicity tests are showed in Fig. 3 for experimental sets (A-F).

In control test, 96% seeds of *L.sativa* were germinated. This indicated that seeds in the present study were viable. The U.S. EPA (1996) index considers a test viable when at least 65% of the seeds have germinated from control. The highest percentage of germination observed in the experimental set F was 93/8%.

### Sequencing and phylogenetic analysis

**Fig. 4:**
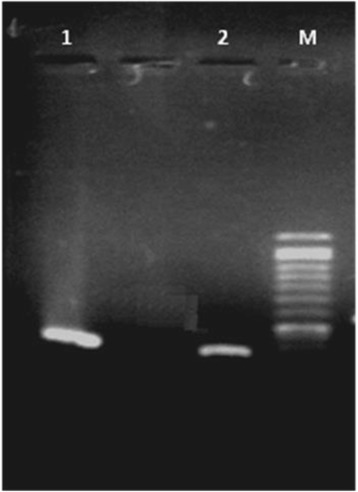
Agarose gel analysis of PCR amplification product using 18S rDNA primers, Line1 and 2 indicates 361 bp size amplicon; M Marker

In order to use SMC containing white-rot fungi successfully for bioremediation, knowledge must be taken from fungal molecular biology. Partial 18 s rDNA sequencing was used for the molecular identification of the *Pleurotus spp*. Figure 4 showed agarose gel electrophoresis separation of PCR product yielded a single band with an approximate size of 361 bp due to the design of specific primer at conserved region. The gene for 18S rRNA displays several traits particularly useful for phylogenetic reconstruction as it contains relatively conserved core segments.

Following PCR amplification, the obtained 361 bp 18S rDNA nucleotide sequence was compared with available 18S ribosomal sequences in the NCBI database using BLASTN. This strain has been registered into a cluster containing *Pleurotus spp*. and exhibited high sequence similarity (100%) to that of *Pleurotus ostreatus* strain Po-13. Hence it was designated as *Pleurotus ostreatus* MFM1. This nucleotide sequence was submitted to a GenBank with accession number KX427171.

## Discussion

Biodegradation with white-rot fungi is one of the newest ways of removing soil contaminants especially petroleum hydrocarbons. In this study, different doses of spent mushroom compost (SMC) of *Agaricomycetes* (wide edible mushroom) were examined for the removal of petroleum hydrocarbons from contaminated soil in the presence or absence of soil enriching compounds.

The results showed that the amount of petroleum hydrocarbons decreased in all contaminated soil samples and decreased further by increasing the volume of SMC to the contaminated soil. The pair wise comparison of the test series also did not identified that petroleum hydrocarbons were further reduced in soil samples enriched with NPK in addition to SMC. There is only a statistically significant difference between removal of petroleum hydrocarbons in the experiments E and F after three months of treatment. Statistical analysis indicated a significant difference between removal of petroleum hydrocarbons from contaminated soil samples after first and second month. Thus proving the efficient role of SMC to biodegradation of Agaricomycetes hydrocarbons (*p* < 0.001). In the experiments A to D, SMC efficiency is increasing from first month to the second, while it is decreased from second month to the third. It seems that inoculation volume of SMC in the experiments A to D is not suitable. During the first and second months, white rot fungi (*P.osteratus*) of SMC have been able to degrade petroleum hydrocarbons, but in third month, because of insufficient of inoculums, degradation fallen. Probably due to the small amount of straw (Due percent less SMC) and disappearance of pores, after 2 months, suitable aerobic conditions for growing mushroom mycelia were not provided. So the destruction of petroleum hydrocarbons has decreased. In the experiments E and F, inoculation volume of SMC has increased to 10%. So it seems with the increase inoculums volume of SMC, requirements for mycelia growth of the fungus in the soil was provided. The bioremediation increased by growth of mycelia fungi. Our results agree with the finding of Zitte et al. who noted that SMC of *P.ostreatus* could be used to amend a diesel contaminated site and they showed a rapid decrease in TPH (90%) in four weeks [19]. Ekundayo reported that *P.ostreatus* reduced the initial total hydrocarbons content to 8% and 9% in soils contaminated with 20% of crude and engine oils, respectively, which was lower than that of *P. pulmonarius*. He showed these mushroom increased the organic carbon, nitrogen and phosphorus contents in contaminated soils after six months [20]. Perhaps that is why in our study the bioremediation of petroleum hydrocarbons in the presence or absence of NPK makes no difference.

The sequences were also investigated using Chromas. Comparison of the amplified fragment (18 s rDNA) sequence of *Pleurotus* strain with the BLAST international online tool in the GenBank confirmed that the strain belonged to *P.ostreatus* species with a high homology of 100%. Then the phylogenetic tree was constructed using the amplified sequence and other gene sequences obtained from the GenBank.

The high rate of biodegradation in petroleum hydrocarbons contaminated soils treated by SMC of *P. ostreatus* was observed after three months in the presence of NPK (64.7%). Cavazos-Arroyo et al. demonstrated that SMC of *P. ostreatus* reduced an agricultural soil contaminated with diesel in highest biodegradation rate (72%).

As shown in Fig. 2(a-c), no significant difference existed in the examined soil between NPK-enriched and NPK-free soil samples in terms of petroleum hydrocarbons removal. However, in test series of E and F with higher volume of SMC added to the soil, the removal of petroleum contamination increased significantly in NPK-enriched soil by increasing treatment time for three months. This could arise from the high number of existing indigenous microorganisms in contaminated soils which participate in a consortium along with SMC mushroom for degradation of petroleum hydrocarbons [21, 22]. It is obvious that the addition of NPK to the soil can help this consortium grow better [23]. Ogbo et al. reported that the addition of NPK to crude oil contaminated soil in the presence of *P. tuberregium* affected the soil negatively [24].

There was a significant difference in all test series (A-F) between the removal of soil contaminated with petroleum hydrocarbons and the control sample (*p* < 0.001). This observation confirms the fact that the mycelia of *Pleurotus* in SMC are involved in the biodegradation of petroleum hydrocarbons in soil [25].

Another important issue is that the bulk of edible mushroom compost is comprised of straw, a large amount of which remains intact in the spent compost [26]. Therefore, addition of SMC to petroleum-contaminated soils, with mostly a clay form, is very convenient, because it creates many pores in the soil and makes possible the growth of a variety of aerobic microorganisms and fungi.

SMC takes a soil-like state after three months and the smell of soil contaminated with petroleum hydrocarbons changes. The ecotoxicity test also showed that adding 10% SMC along with NPK greatly reduces the contamination of petroleum hydrocarbons in soil after 3 months.

It is important to identify potential petroleum hydrocarbons on different terrestrial ecosystems. The increases of the petroleum hydrocarbons in soil decreased the germination index. The lowest percent of germination was obtained from soil extracts contaminated with petroleum hydrocarbons with 3% SMC. Ecotoxicity tests were performed in 6 series, and the results showed that the germination percentage increased in all cases of NPK-added experiments compared with NPK-free experiments.

The results related to the percentage of petroleum hydrocarbons removal from contaminated soil and the germination rate completely confirm each other [7, 27]. These experiments show that increased volume of SMC to soil contaminated with petroleum hydrocarbons can effectively reduce the amount of these petroleum compounds in contaminated soils. Also adding enrichment compounds such as NPK or other organic fertilizers have a very important role in advancing this process [28].

## Conclusions

Bioremediation using composting is a hopeful technology. *Agaricomycetes* has applied as not only favorable technology for the bioremediation of hydrocarbon contaminated soils, but also as a totally environmentally friendly technique. The results of this study had clearly shown that with the addition of 10% of the SMC of *Pleurotus ostreatus* can degrade petroleum contaminated soil over a short period of time. Add NPK hasn't significantly effect to increase the rate of mycoremediation. However, bioremediation increase by increasing of SMC percentage.

## Abbreviations

PDA: Potato dextrose agar
SDS: Sodium dodecyl sulfate
SMC: Spent mushroom compost

## References

[1] Pinedo J, Ibáñez R, Irabien Á (2014). A comparison of models for assessing human risks of petroleum hydrocarbons in polluted soils. Environ Model Software.

[2] Djelal H, Amrane A (2013). Biodegradation by bioaugmentation of dairy wastewater by fungal consortium on a bioreactor lab-scale and on a pilot-scale. J Environ Sci.

[3] Harms H, Schlosser D, Wick LY (2011). Untapped potential: exploiting fungi in bioremediation of hazardous chemicals. Nat Rev Micro.

[4] Mazaheri Assadi M, Ardeshiri M, Sheykhzadeh H, Jahangiri M (2014). The bioremediation of crude oil contaminated soil. Pet Sci Technol.

[5] Anastasi A, Varese GC, Bosco F, Chimirri F, Marchisio VF (2008). Bioremediation potential of basidiomycetes isolated from compost. Bioresource Technol.

[6] Adenipekun CO, Fasidi IO (2008). Bioremediation of oil-polluted soil by Lentinus subnudus, a Nigerian white-rot fungus. Afr J Biotechnol.

[7] Okerentugba P, Orji F, Ibiene A, Elemo G (2015). Spent mushroom compost for bioremediation of petroleum hydrocarbon polluted soil: A review. J Environ Sci Toxicol.

[8] Thapa B, Kumar KC, Ghimire A (2012). A review on bioremediation of petroleum hydrocarbon contaminants in soil. J Sci EnginTechnol.

[9] Adenipekun C, Lawal R (2012). Uses of mushrooms in bioremediation: A review. Biotechnol Mol Biol Rev.

[10] Environmental Protection Agency U. Total petroleum hydrocarbons: SW846 method 8100 modified/. United State Environmental Protection Agency; 2008. p. 2–23.

[11] Wilke B. Determination of Chemical and Physical Soil Properties(Soil Dry Mass andWater Content). Manual for Soil Analysis. Berlin: 5: Springer-Verlag Berlin Heidelberg; 2005. p. 47–9.

[12] Dochukwu U, Udinyiwe O, Adeghe O, Omeje F (2014). Comparative effects of mashed mushroom and N.P.K Fertilizer on the bioremediation of crude oil polluted soil. Int J Curr Microbiol App Sci.

[13] Chorom M, Sharifi H, Motamedi H (2010). Bioremediation of a crude oil - polluted soil by application of fertilizer. J Env Health Sci Eng.

[14] Márquez-Rocha FJ, Hernández-Rodrí V, Lamela MT (2001). Biodegradation of Diesel oil in soil by a microbial consortium. Water Air Soil Pollut.

[15] Cruz J, Lopes P, Montagnolli R, Tamada I, Guerra Silva N, Bidoia E (2013). Toxicity assessment of contaminated soil using seeds as bioindicators. J Appl Biotechnol.

[16] Hentati O, Lachhab R, Ayadi M, Ksibi M (2013). Toxicity assessment for petroleum-contaminated soil using terrestrial invertebrates and plant bioassays. Environ Monit Assess.

[17] Al-Samarrai TH, Schmid J (2000). A simple method for extraction of fungal genomic DNA. Lett Appl Microbiol.

[18] Dentinger BTM, Margaritescu S, Moncalvo J-M (2010). Rapid and reliable high-throughput methods of DNA extraction for use in barcoding and molecular systematics of mushrooms. Mol Ecol Res.

[19] Zitte L, Awi-Waadu G, John A (2012). Effect of oyster mushroom (Pleurotus ostreatus) mycelia on petroleum hydrocarbon contaminated substrate. J Agri Social Res.

[20] Ekundayo F (2014). Comparative studies on biodegradative abilities of Pleurotus ostreatus and P. pulmonarius in soils contaminated with crude and used engine oils. Adv Microb.

[21] Mazaheri Assadi M, Tabatabaee MS (2010). Biosurfactants and their use in upgrading petroleum vacuum distillation residue: a review. Int J Environ Res.

[22] Akhavan Sepahi A, Dejban Golpasha I, Emami M, Nakhoda A (2008). Isplation and characterization of crude oil degrading Bacillus spp. J Env Health Sci Eng.

[23] Gasecka M, Drzewiecka K, Stachowiak J, Siwulski M, Golin´ski P, Sobieralski K (2012). Degradation of polycyclic aromatic hydrocarbons (PAHs) by spent mushroom substrates of Agaricus bisporus and Lentinula edodes. Acta Sci Pol-Hortoru.

[24] Ogbo EM, Okhuoya JA (2009). Effect of crude oil contamination on the yield and chemical composition of pleurotus tuberregium. Afr J Food Sci.

[25] Emuh FN (2010). Mushroom as a purifier of crude oil polluted soil. Int J Sci Nat.

[26] Lau KL, Tsang YY, Chiu SW (2003). Use of spent mushroom compost to bioremediate PAH-contaminated samples. Chemosphere.

[27] Phan C-W, Sabaratnam V (2012). Potential uses of spent mushroom substrate and its associated lignocellulosic enzymes. Appl Microbiol Biotechnol.

[28] Tabatabaee MS, Mazaheri Assadi M (2013). Vacuum distillation residue upgrading by an indigenous bacillus cereus. J Environ Health Sci Eng.

